# Factors associated with surgeon recommendation for additional cast immobilization of a CT-verified nondisplaced scaphoid waist fracture

**DOI:** 10.1007/s00402-021-04062-0

**Published:** 2021-07-24

**Authors:** Anne Eva J. Bulstra, Tom J. Crijns, Stein J. Janssen, Geert A. Buijze, David Ring, Ruurd L. Jaarsma, Gino M. M. J. Kerkhoffs, Miryam C. Obdeijn, Job N. Doornberg, The Science of Variation Group, A. Peters, A. B. Spoor, Abhay Shrivastava, Aakash Chauhan, Adam Shafritz, Asif M. Ilyas, Anne J. H. Vochteloo, Andrew John Powell, Alberto Pérez Castillo, Alexandre Leme Godoy-Santos, Amparo Gomez Gelvez, Andrea Bauer, Antonio Barquet, Anze Kristan, Ante Prkic, Axel Jubel, Boj Mirck, B. E. Kreis, H. Brent Bamberger, William Dias Belangero, Bernard F. Hearon, Bradley Palmer, Brad Hyatt, Brian P. D. Wills, Henry Broekhuyse, Richard Buckley, Burak Altintas, Sean T. Campbell, Carl Ekholm, Carlos Henrique Fernandes, C. H. Fernandes, Carl Weiss, Christos Garnavos, Charles Metzger, Christopher J. Wilson, Chris Bainbridge, Christian Deml, Jesus Moreta, Conor Kleweno, Constanza L. Moreno-Serrano, Craig B. Ordway, Cyrus Klostermann, David Zeltser, David G. Dennison, Diederik O. Verbeek, Dan Polatsch, Camilo Jose Romero Barreto, Koroush Kabir, Mohamed Shafi, Juan M. Patiño, Roger van Riet, Samir Sodha, Scott Duncan, Daniel C. Wascher, Edward F. Ibrahim, Efstathios G. Ballas, Edward Harvey, Edward K. Rodriguez, Emilia Stojkovska Pemovska, E. Walbeehm, Peter J. Evans, Ezequiel E. Zaidenberg, Fred O’Brien, Franz Josef Seibert, Frank W. Bloemers, Gladys Cecilia Zambrano Caro, Gregory DeSilva, George Babis, George Pianka, Michael Githens, Giselly Miranda Veríssimo, Grant E. Garrigues, Guido Fierro, Holger Durchholz, Jeremy Hall, Hal McCutchan, Michael Nancollas, Colby Young, Greg P. Watchmaker, Gary M. Pess, Lewis B. Lane, Harold Alonso Villamizar, Ippokratis Pountos, Hervey L. Kimball, Eric P. Hofmeister, Iain McGraw, Konul Erol, J.F. Di Giovanni, Jacob W. Brubacher, Jan Biert, Jason C. Fanuele, Jason D. Tavakolian, Jack Choueka, Jose Eduardo Grandi Ribeiro, Jose Eduardo Grandi Ribeiro Filho, Joseph M. Conflitti, J. M. R Roiz, John Munyak, James F. Nappi, Job N. Doornberg, John M. Erickson, Jorge G. Boretto, Joel M. Post, Jorge Rubio, John A. Scolaro, John Taras, Julio Domenech, Julio Sandoval, Jeffrey Wint, Katherine Celeste Faust, Ken Butters, Kyle Jeray, Karl-Josef Prommersberger, Kagan Ozer, G. A. Kraan, Kyle J. Chepla, L. M. S. J. Poelhekke, Ladislav Mica, Lawrence Weiss, Lars Adolfsson, Lars C. Borris, Louis Christopher Grandizio, Leon Elmans, Luis Felipe Náquira Escobar, L. W. van der Plaat, M. Verhofstad, Marcos Sanmartin-Fernandez, Mario Di Micoli, Matej Kastelec, Maurizio Calcagni, Max Talbot, Maarten W. G. A. Bronkhorst, John A. McAuliffe, Michael Behrman, M. Quell, Michael Nakashian, Minoo Patel, Matthew Bengard, M. Jason Palmer, Michael Prayson, Matthias Knobe, Marinis Pirpiris, Minos Tyllianakis, Michael W. Grafe, Neal Chen, Nelson Elias, Ngozi M. Akabudike, Nathan A. Hoekzema, Nicholas L. Shortt, Nikolaos Kanakaris, Nikolas H. Kazmers, Nina Lightdale-Miric, Jim Calandruccio, Ole Brink, Martin Richardson, Jose A. Ortiz Jr, Pascal F. W. Hannemann, P. V. van Eerten, Prashanth Inna, Peter Althausen, Panagiotis Lygdas, Nata Parnes, Paul A. Martineau, Prosper Benhaim, Philip Forno, Pradeep Choudhari, Peter Hahn, Peter Fedele Townsend, Peter Giannoudis, Paul Guidera, Philipp Muhl, Philipp Streubel, Peter Jebson, Patrick W. Owens, Tamir Pritsch, Paul J. Scibetta Jr., Rob Nelissen, Robert Haverlag, R. H. van Leerdam, Ramon de Bedout, Sergio Rowinski, Reid W. Draeger, R. Fricker, Richard Wallensten, Richard S. Gilbert, Marco Rizzo, Richard Jenkinson, Robert E. Van Demark Jr, Craig Rodner, Rachel S. M. D. Rohde, Richard S. Page, David Ruch, Vani J. Sabesan, Stephen A. Kennedy, Niels W. L. Schep, Scott Mitchell, Sebastian Farr, Paul A. D. O. Sibley, Scott G. Kaar, S. A. Meylaerts, Steven L. Henry, Steven Meletiou, Steven J. Morgan, Marc Swiontkowski, T. Schepers, Thomas DeCoster, Taizoon Baxamusa, F. Thomas D. Kaplan, Thierry Begue, Thomas Mittlmeier, Thomas Rebele, T Apard, Tim Chesser, Tomo Havlifçek, T. Rozental, Theodoros Tosounidis, H. Utkan Aydin, Vincenzo Giordano, Varun Kashyap Gajendran, Vasileios S. Nikolaou, Vincent Ruggiero, Warren C. Hammert, Yoram Weil, Wojciech Satora, Zsolt Balogh

**Affiliations:** 1 Department of Orthopedic and Trauma Surgery, Flinders Medical Centre, Flinders University, Flinders Drive, Bedford Park, Adelaide, South Australia 5042 Australia; 2 Amsterdam UMC, Location AMC, Department of Orthopedic Surgery, University of Amsterdam, Meibergdreef 9, 1105 AZ Amsterdam, The Netherlands; 3 Department of Surgery and Perioperative Care, Dell Medical School, Health Discovery Building, The University of Texas at Austin, 6706, 1701 Trinity Street, Austin, TX78712 USA; 4 Clinique Générale Annecy, Hand and Upper Limb Surgery, 4, Chemin de La Tour La Reine, 74000 Annecy, France; 5 Department of Plastic, Reconstructive and Hand Surgery, Amsterdam UMC, Location AMC, University of Amsterdam, Meibergdreef 9, 1105 AZ Amsterdam, The Netherlands

**Keywords:** Scaphoid, Fracture, Cast, Immobilization, Decision-making

## Abstract

**Introduction:**

Data from clinical trials suggest that CT-confirmed nondisplaced scaphoid waist fractures heal with less than the conventional 8–12 weeks of immobilization. Barriers to adopting shorter immobilization times in clinical practice may include a strong influence of fracture tenderness and radiographic appearance on decision-making. This study aimed to investigate (1) the degree to which surgeons use fracture tenderness and radiographic appearance of union, among other factors, to decide whether or not to recommend additional cast immobilization after 8 or 12 weeks of immobilization; (2) identify surgeon factors associated with the decision to continue cast immobilization after 8 or 12 weeks.

**Materials and methods:**

In a survey-based study, 218 surgeons reviewed 16 patient scenarios of CT-confirmed nondisplaced waist fractures treated with cast immobilization for 8 or 12 weeks and recommended for or against additional cast immobilization. Clinical variables included patient sex, age, a description of radiographic fracture consolidation, fracture tenderness and duration of cast immobilization completed (8 versus 12 weeks). To assess the impact of clinical factors on recommendation to continue immobilization we calculated posterior probabilities and determined variable importance using a random forest algorithm. Multilevel logistic mixed regression analysis was used to identify surgeon characteristics associated with recommendation for additional cast immobilization.

**Results:**

Unclear fracture healing on radiographs, fracture tenderness and 8 (versus 12) weeks of completed cast immobilization were the most important factors influencing surgeons’ decision to recommend continued cast immobilization. Women surgeons (OR 2.96; 95% CI 1.28–6.81, *p*  =  0.011), surgeons not specialized in orthopedic trauma, hand and wrist or shoulder and elbow surgery (categorized as ‘other’) (OR 2.64; 95% CI 1.31–5.33, *p*  =  0.007) and surgeons practicing in the United States (OR 6.53, 95% CI 2.18–19.52, *p*  =  0.01 versus Europe) were more likely to recommend continued immobilization.

**Conclusion:**

Adoption of shorter immobilization times for CT-confirmed nondisplaced scaphoid waist fractures may be hindered by surgeon attention to fracture tenderness and radiographic appearance.

## Introduction

Evidence from clinical trials suggests that a scaphoid waist fracture that is nondisplaced on computed tomography (CT) will heal with adequate immobilization [1–5]. Screw fixation helps people with a nondisplaced waist fracture avoid cast wear, but it does not improve long-term outcomes [6–8]. A shorter period of immobilization may reduce the perceived benefits of operative treatment [6]. In the absence of a second injury, the probability of nonunion for a CT- or MRI-confirmed nondisplaced scaphoid waist fracture is below 1% [1–5, 9]. Among five clinical prospective and one retrospective series that used CT or MRI to diagnose displacement, only two in 362 (0.6%) of the nondisplaced waist fractures treated with cast immobilization did not heal [1–5, 9]. It is not clear whether the diagnosis of nonunion in these two fractures was based on imaging 4–12 weeks after injury, or also confirmed radiologically 6 months or more after injury [3, 4]. Radiological diagnosis of union is unreliable on radiographs and is of questionable reliability on CT within 3–4 months after injury [10–12]. It is also possible that at least one of these fractures was displaced as it demonstrated moderate translation on the 4-week CT scan and there was no CT scan at the time of injury [4].

The improved understanding of the link between displacement and nonunion has led some to consider shorter (less than the conventional 8–12 weeks) and less rigid (e.g., thumb free) types of immobilization for CT-confirmed nondisplaced scaphoid waist fractures [1, 3–5, 13]. Some have tested immobilization of CT- or MRI-confirmed nondisplaced fractures with as few as 4–6 weeks of immobilization with good results in preliminary trials.[4]

In our experience, the concepts leading some to consider a shorter immobilization duration for nondisplaced scaphoid waist fractures conflict with the fact that (1) radiographic appearance of union, and (2) tenderness at the fracture site upon physical examination (fracture tenderness) are often used to decide whether to continue cast immobilization. These traditional concepts run counter to lines of evidence that (1) diagnosis of scaphoid fracture union on radiographs is unreliable [11, 12] and (2) patient-reported pain intensity, including fracture tenderness [14], is strongly related to patient psychosocial factors including cognitive biases about pain and coping strategies in patients with upper extremity injury [15–17].

Based on studies reporting near 100% of the CT-confirmed nondisplaced waist fractures heal and that radiographs and examination are unreliable and inaccurate for diagnosis of union, one can argue that using fracture tenderness and radiographic appearance to recommend additional cast wear after 8 weeks of immobilization may lead to unhelpful and potentially harmful overtreatment in a substantial proportion of patients. To reduce immobilization time, surgeon decision making would need to evolve to match the existing evidence. One can therefore argue that surgeons may need to accept the uncertainty about radiographic appearance and fracture tenderness.

This study aimed to identify (1) what proportion of surgeons recommends additional cast immobilization of a CT-verified nondisplaced scaphoid waist fracture after 8 and 12 weeks of cast wear; (2) what clinical variables (patient sex, age, healing on radiographs, fracture tenderness, duration of cast wear completed) are associated with surgeon recommendation to continue immobilization of a nondisplaced scaphoid waist fracture after 8 and 12 weeks and (3) what surgeon variables (sex, location of practice, subspeciality, years in practice) are associated with surgeon recommendation to continue immobilization of a nondisplaced scaphoid waist fracture after 8 and 12 weeks.

## Methods

### Patient scenarios

Sixteen scenarios of patients with a nondisplaced scaphoid waist fracture were presented to orthopedic surgeons, (European) trauma surgeons that treat scaphoid fractures, and (plastic) hand- and wrist surgeons. Scenarios contained brief descriptions of patients with a CT-confirmed nondisplaced scaphoid waist fracture, treated non-operatively with 8 or 12 weeks of cast immobilization. Surgeons were asked whether they would recommend to continue cast immobilization. For each case scenario, the following five clinical **(**patient) variables varied: (1) sex, (2) description of fracture healing on radiographs (clear versus unclear healing), (3) presence of fracture tenderness (minimal to none versus notable), (4) duration of completed cast immobilization (8 versus 12 weeks) and patient age, randomly generated between 18 and 32 years and 43–57 years. SurveyMonkey (Palo Alto, CA, USA) was used to create an online survey. The vignettes were presented in random order.

### Participants (surgeons)

We invited members of the ‘Science of Variation Group’ (SOVG) to participate in this web-based study. The SOVG is an international web-based collaboration of orthopedic, trauma and hand and wrist surgeons, set out to investigate the variation in interpretation, classification and treatment of illness among surgeons through web-based experiments [18]. The SOVG provides no other incentive for participation than group authorship or acknowledgement, depending on the publishing Journal.

A total of 225 surgeons participated. Seven respondents that were residents (physicians in training) were excluded, leaving 218 participants for analysis. Participating surgeon demographics are summarized in Table 1.

**Table 1:** Participating surgeon characteristics

	*n*	%
Sex		
Male	205	94
Female	13	6
Location of practice		
United States	102	47
Europe	75	34
Other	41	19
Subspecialty		
Hand and wrist	93	43
Orthopedic trauma	67	31
Shoulder and elbow	30	14
Other	28	13
Years in practice		
0–5	61	28
6–10	50	23
11–20	66	30
21–30	41	19
Supervising trainees		
Yes	175	80
No	43	20

*n* number of participating surgeons

### Statistical methods

Descriptive analysis was performed, reporting the number of recommendations for continued cast immobilization per patient scenario. We pooled surgeon practice location as ‘Other’ for surgeons practicing outside the United States or Europe.

To assess the impact of clinical (patient) factors on surgeon recommendation to continue cast immobilization we used two approaches: (1) posterior probabilities were calculated [19] and (2) variable importance was determined using a random forest algorithm [20].

Posterior probabilities were calculated using Bayes’ theorem. First, the case scenario dta were pooled to calculate the unadjusted probabilities for recommending continued cast immobilization for each included patient variable: age, sex, radiographic fracture healing, fracture tenderness and duration of cast immobilization completed. The unadjusted probability was calculated as the percentage of cases in which surgeons recommended continued immobilization in the presence of each variable. Posterior probability describes the conditional probability of an event occurring, in the presence of a combination of variables, by incorporating the associated probabilities of each of the variables [19]. The resulting posterior probability represents the probability of continuing cast immobilization given the combination of factors and is represented as a percentage. A posterior probability of 100% indicates that participants uniformly agree to continue cast immobilization, a posterior probability of 0% indicates that participants uniformly agree to discontinue immobilization [19].

A random forest algorithm was applied to rank the “importance” of each patient variable [20]. Random forest is a supervised machine learning algorithm that is mostly used for prediction. It is a decision tree-based model that involves repetitive partitioning of a given dataset into two groups until optimized. The variable importance indicates the improvement in functioning of the model based on the included variable; the variable importance score is normalized to the most important variable having an importance score of one [20].

To identify surgeon variables associated with surgeon recommendation for continued cast immobilization, multilevel logistic mixed regression models were constructed. Random intercepts were chosen at the surgeon level. Odds ratio, 95% confidence interval, standard error, random-effects estimate, and p-values are reported. All two-tailed *p* values  <  0.05 were considered statistically significant. Reference values were chosen so that odds ratios were greater than one.

An ante-hoc sample size calculation demonstrated a minimum sample size of 90 participants to provide 80% statistical power (beta  =  0.20; two-tailed alpha  =  0.05) to detect a medium effect size of 0.3, using a paired *t* test.

## Results

### Proportion of surgeons recommending additional cast immobilization

The proportion of surgeons recommending continued cast immobilization after 8 weeks of cast wear averaged 47% (range: 10–84%) depending on patient characteristics. After 12 weeks of immobilization the proportion of surgeons recommending additional cast wear averaged 21% (range 2–49%; Table 2).

**Table 2:** Patient scenario characteristics and surgeon recommendation to continue cast immobilization

Patient scenario number	Age (years)	Sex	Fracture healing on radiograph	Fracture tenderness	Cast duration (weeks)	Surgeons recommending to continue cast immobilization	
						*n*	%
2	22	Female	Unclear	Yes	8	178	84
10	48	Male	Unclear	Yes	8	169	80
4	20	Female	Unclear	No	8	131	62
12	56	Male	Unclear	No	8	115	54
1	23	Female	Unclear	Yes	12	103	49
9	52	Male	Unclear	Yes	12	92	43
6	57	Female	Clear	Yes	8	84	39
14	23	Male	Clear	Yes	8	83	39
11	31	Male	Unclear	No	12	51	24
3	51	Female	Unclear	No	12	46	22
13	49	Male	Clear	Yes	12	31	15
5	18	Female	Clear	Yes	12	27	13
8	57	Female	Clear	No	8	23	11
16	23	Male	Clear	No	8	21	9.9
7	46	Female	Clear	No	12	5	2.3
15	19	Male	Clear	No	12	5	2.3

*n* number of surgeons

### Clinical (patient) variables associated with surgeon recommendation for additional immobilization

**Fig. 1:**
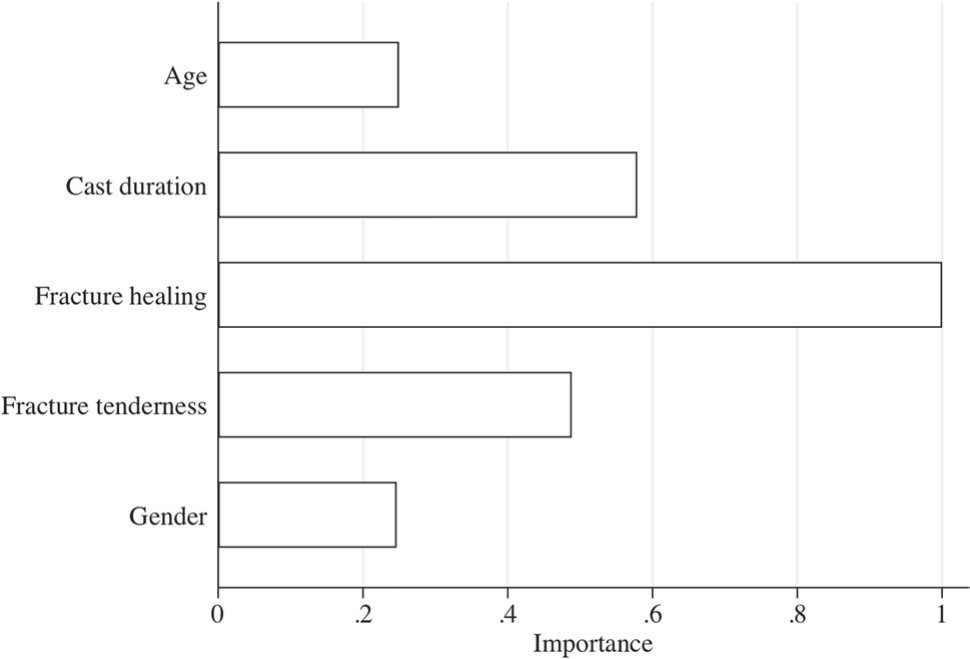
random forest variable importance score of predictor patient variables for surgeon recommendation to continue or not continue cast immobilization. Ranked importance score of each patient variable as a predictor for surgeon recommendation to continue cast immobilization. The variable importance score is normalized to the most important variable having an importance score of one

Appearance of fracture healing on radiographs, fracture tenderness, and duration of cast immobilization were the most important factors when recommending additional cast immobilization or not. Based on posterior probabilities, we found that a combination of unclear fracture healing on radiographs, the presence of notable fracture tenderness, and 8 weeks (versus 12 weeks) of cast immobilization yielded the highest posterior probability of surgeon recommendation to continue cast immobilization (range 73–76%). The lowest posterior probability was yielded in cases with clear radiographic fracture healing, no fracture tenderness, and 12 weeks of immobilization completed (6%; Table 3). Random forest analysis demonstrated that the most predictive factors for recommending to continue cast immobilization or not were in order of importance: radiographic fracture healing, duration of cast immobilization, and fracture tenderness; followed by age and sex which were of equal importance (Fig. 1).

**Table 3:** Patient variables: posterior probability of surgeon recommendation to continue cast immobilization

Age (years)	Sex	Fracture healing on radiograph	Fracture tenderness	Cast duration (weeks)	Posterior probability^a^ (%)
< 35	Female	Unclear	Yes	8	76
> 35	Male	Unclear	Yes	8	73
< 35	Female	Unclear	No	8	54
< 35	Male	Clear	Yes	8	51
> 35	Male	Unclear	No	8	50
< 35	Female	Unclear	Yes	12	49
> 35	Male	Unclear	Yes	12	45
> 35	Female	Clear	Yes	8	35
< 35	Male	Unclear	No	12	25
> 35	Female	Unclear	No	12	25
< 35	Male	Clear	No	8	17
> 35	Female	Clear	No	8	16
< 35	Female	Clear	Yes	12	15
> 35	Male	Clear	Yes	12	13
< 35	Male	Clear	No	12	5.6
> 35	Female	Clear	No	12	5.6

^a^The posterior probability of surgeons recommending to continue cast immobilization is defined as the probability of a surgeon recommending to continue cast immobilization in the presence of five defined variables, taking into account the unadjusted probability to continue cast immobilization of each variable.

### Surgeon variables associated with surgeon recommendation for additional immobilization

Multilevel logistic mixed regression analysis identified that surgeons not specialized in hand and wrist surgery, shoulder and elbow or orthopedic trauma (categorized as ‘Other’) were more likely to recommend longer cast wear compared to hand and wrist surgeons. Surgeons practicing outside of Europe (i.e., United States or ‘Other’) were significantly more likely to continue cast wear compared to surgeons practicing in Europe. Female surgeons were more likely to continue cast immobilization compared to male surgeons. Years of practice or whether surgeons supervised trainees, were not associated with surgeon recommendation to continue cast immobilization (Table 4).

**Table 4:** Multilevel logistic regression analysis of surgeon variables associated with surgeon recommendation to continue cast immobilization

	Odds ratio	95% CI			Standard error	*p* value	Random-effects estimate (95% CI)	Standard error
							2.1 (0.82–5.38)	1.0
Sex								
Male	Reference value							
Female	2.96	1.28–6.81			1.26	0.011*		
Years in practice								
0–5	1.61	0.97–2.66			0.41	0.064		
6–10	Reference value							
11–20	1.09	0.72–1.66			0.23	0.668		
21–30	1.49	0.90–2.46			0.38	0.119		
Location of practice								
Europe	Reference value							
United States	6.53		2.18–19.52		3.65	0.001*		
Other	4.22		1.71–10.38		1.94	0.002*		
Supervising trainees								
Yes	Reference value							
No	1.11			0.76–1.60	0.21	0.593		
Subspecialty								
Hand and wrist	Reference value							
Orthopedic trauma	1.05			0.73–1.52	0.20	0.785		
Shoulder and elbow	1.31			0.81–2.10	0.32	0.266		
Other	2.64			1.31–5.33	0.95	0.007*		

*95% CI *95% confidence interval

^*^Significant at *p * <  0.05

## Discussion

An increasing number of studies is considering immobilization times less than the conventional 8–12 weeks for the treatment of CT-confirmed nondisplaced scaphoid waist fractures [1, 2, 4]. The decision to continue immobilization is often based on radiographs and fracture tenderness. This conflicts with evidence that radiographs are unreliable to diagnose scaphoid union [10–12] and that pain intensity is strongly correlated to coping strategies in response to nociception in patients with upper extremity injury [14–17]. The discrepancy between current evidence and surgeon-decision making may result in unhelpful additional immobilization. This study investigated clinical (patient) and surgeon variables associated with surgeon decision to continue cast immobilization after 8 or 12 weeks.

This study has several limitations. It is possible that some surgeons interpreted the decision against additional immobilization as representing the option to perform surgery instead. We introduced the scenario as a patient with a nearly 100% likelihood of union with nonoperative treatment. It is notable that at least one surgeon considered surgery an option when choosing not to continue immobilization and contacted us. Based on comments and observed trends in recommendations most surgeons appear to have understood that the survey was not positing surgery as an option. Second, case descriptions can only approximate clinical encounters. To allow for statistical analysis, we studied five patient factors. We did not study presentation delay, mechanism of injury, or profession. Also, surgeons were given the option to continue cast immobilization. Options such as removable splints were not included. Furthermore, surgeons were presented with a description of a radiograph, rather than an actual radiograph. Since we were interested in the effect of radiographic union on decision-making—and not surgeons’ individual radiographic interpretations—this was done deliberately to avoid noise from the unreliability of radiographic interpretation of union. Only 13 out of 218 surgeons were women and our findings may not be representative of all female surgeons. The finding that women were more likely to continue immobilization is contradictory to findings by Paulus et al. [21] and may be spurious.

On average, 47% and 21% of the surgeons recommended continued immobilization of a nondisplaced scaphoid waist fracture after 8 and 12 weeks of completed cast wear, respectively. Traditionally, cast immobilization has been prescribed for 8–12 weeks [22–24]. More recent studies have investigated immobilization as short as 4–6 weeks for CT-verified nondisplaced waist fractures [2, 4]. Geoghegan et al. [4] allowed patients with scaphoid waist fractures to mobilize if their fracture appeared united and nondisplaced on a 4-week CT scan. All such fractures united. All but one of the remaining nondisplaced waist fractures healed with 5–8 weeks of immobilization. The one fracture that was reported as ununited showed moderate translation on the 4-week CT and may have been displaced [4]. Studies implementing shorter cast duration regardless of radiographic appearance at 4–12 weeks, or randomized controlled trials comparing less than or more than 8 weeks of immobilization are lacking. This and the limited reliability of radiographs [11, 12] or CT [10] to diagnose nonunion within 4 months after injury create a situation of uncertainty and room for patients to express their preferences regarding the various treatment approaches. A return appointment to document union after 6 months could be considered.

Radiographic appearance of union, fracture tenderness and the duration of cast immobilization were the most important clinical factors affecting surgeon recommendation for additional immobilization. Nearly half the patients immobilized for 8 weeks and a fifth of patients immobilized for 12 weeks were recommended to continue immobilization if radiographic union was “unclear”. This runs counter to good evidence that radiographs have poor to moderate reliability in assessing scaphoid union and are inaccurate at diagnosing nonunion [11, 12]. CT scans are considered more reliable and accurate than radiographs to assess union by some [25]. Caution is warranted however, even when relying on CT to assess union. The low prevalence of nonunion in CT-confirmed nondisplaced waist fractures, makes the diagnosis of non- or delayed union more likely to be inaccurate within 4 months after injury, even with CT [10]. Importantly, Buijze et al. confirmed union on 24-week radiographs, in all patients with a CT-confirmed nondisplaced waist fracture, whose immobilization was discontinued after 10 weeks despite ‘incomplete’ (<  25% trabecular bridging) or no signs of healing on a 10-week CT scan [5]. As such, it is not clear whether the appearance of a scaphoid fracture on CT 6–12 weeks after injury is associated with a benefit from additional immobilization. This suggests that a shorter duration of immobilization will only be possible if surgeons are influenced less by radiographic appearance and rely more on the evidence that a CT-confirmed nondisplaced scaphoid waist fracture is very likely to heal no matter the radiographic appearance 12 weeks after injury.

Fracture tenderness also led to additional immobilization. There is considerable evidence that pain intensity is strongly associated with symptoms of depression, anxiety and less effective coping strategies in response to nociception [15–17]. Gonzalez et al. [14] reported a correlation between greater pain on examination and less adaptive responses to pain among 117 people with a healing upper extremity function with no risk of nonunion. This suggests that fracture tenderness may not be a helpful measure of fracture union.

We identified variation in surgeon recommendation to continue immobilization among surgeons of different specialties, regions and sex. This reflects the lack of evidence-based decision-making and may be due to disagreement about optimal cast duration. Differences in medicolegal systems may also play part in this variation. Surgeons practicing in the United States may be more likely to recommend additional immobilization due to the more litigious medicolegal climate compared to Europe. A survey study of 494 international surgeons documented 30%, 33% and 27% of the surgeons recommending 6, 8 or 12 week of cast immobilization respectively, with no variation by specialty [21].

In conclusion, fracture tenderness and radiographic appearance of union have a substantial influence on surgeon recommendation for additional immobilization of a CT-confirmed nondisplaced scaphoid waist fracture, even after 12 weeks of immobilization. Because fractures are likely to have some residual tenderness and equivocal radiological appearance after 8–12 weeks, the continued influence of these factors may result in unhelpful immobilization. To adopt shorter immobilization times, surgeons may need to accept uncertainty regarding fracture tenderness and radiographic fracture appearance and rely more on the evidence suggesting these fractures are very likely to heal, even with relatively brief protection.
